# Therapeutic potential of *Curcuma longa* against monkeypox: antioxidant, anti-inflammatory, and computational insights

**DOI:** 10.3389/fchem.2024.1509913

**Published:** 2025-01-16

**Authors:** Farouk Boudou, Amal Belakredar, Ahcen Keziz, Linda Aissani, Huda Alsaeedi, David Cronu, Mikhael Bechelany, Ahmed Barhoum

**Affiliations:** ^1^ Department of Biology, Faculty of Natural and Life Sciences, Djillali Liabes University of Sidi-Bel-Abbes, Sidi-Bel-Abbes, Algeria; ^2^ Department of Biotechnology, Faculty of Natural Sciences and Life, University of Mostaganem Abdelhamid Ibn Badis, Mostaganem, Algeria; ^3^ Department of Physics, Physics and Chemistry of Materials Laboratory, University of M’sila, M’sila, Algeria; ^4^ Matter sciences Department, Abbes Laghrour- University of Khenchela, Khenchela, Algeria; ^5^ Department of Chemistry, College of Science, King Saud University, Riyadh, Saudi Arabia; ^6^ Institut Européen des Membranes, IEM, UMR-5635, Univ Montpellier, ENSCM, CNRS, Montpellier, France; ^7^ Functional Materials Group, Gulf University for Science and Technology (GUST), Mubarak Al-Abdullah, Kuwait; ^8^ NanoStruc Research Group, Chemistry Department, Faculty of Science, Helwan University, Cairo, Egypt

**Keywords:** Mpox virus, *Curcuma longa*, curcuminoids, antioxidant activity, anti-inflammatory properties, molecular docking, molecular dynamic simulations

## Abstract

**Background:**

Monkeypox (Mpox) is a re-emerging zoonotic disease with limited therapeutic options, necessitating the exploration of novel antiviral agents. *Curcuma longa* (turmeric) is a widely used medicinal plant known for its antioxidant and anti-inflammatory properties, primarily attributed to its bioactive curcuminoids.

**Aim:**

This study aimed to evaluate the therapeutic potential of *C. longa* aqueous extract (CAE) against monkeypox through phytochemical characterization, biological assays, and computational analyses.

**Methodology:**

Phytochemical analysis, including HPLC, identified key Curcumin, Bisdemethoxycurcumin, Demethoxycurcumin, Tetrahydrocurcumin, Curcuminol, and Ar-curcumene. The DPPH assay and total antioxidant capacity (TAC) were employed to assess antioxidant activity. Anti-inflammatory effects were determined by measuring the inhibition of heat-induced protein denaturation. Molecular docking and molecular dynamics (MD) simulations were performed to evaluate the interactions between curcuminoids and monkeypox virus proteins.

**Results:**

The aqueous extract of *C. longa* was prepared via decoction, yielding 7.80% ± 0.81% extract with curcumin as the predominant compound (36.33%). The CAE exhibited strong antioxidant activity with a TAC of 36.55 ± 0.01 µg GAE/g d.w., an IC50 of 0.77 ± 0.04 mg/mL in the DPPH assay, andan EC50 of FRAP of 3.46 ± 0.11 mg/mL. Anti-inflammatory analysis showed 78.88 ± 0.53%inhibition for egg albumin and 90.51 ± 0.29%for BSA. Molecular docking identified demethoxycurcumin (DMC) as the most potent compound, with binding affinities of −8.42 kcal/mol (4QVO), −7.61 kcal/mol (8CEQ), and −7.88 kcal/mol (8QRV). MD simulations confirmed the stability of DMC complexes, with the 4QVO-DMC interaction being the most stable, showing RMSD fluctuations within a range of 0.2–0.6 nm, with an average fluctuation of 0.4 nm, and consistent compactness with Rg values remaining between 1.8 and 2.0 nm, with a fluctuation of only 0.2 nm over 100 ns.

**Discussion:**

The results demonstrate the multifunctional therapeutic potential of *C. longa*, driven by its potent antioxidant and anti-inflammatory properties. The computational findings suggest that curcuminoids, particularly demethoxycurcumin, could serve as promising antiviral agents against monkeypox. These findings pave the way for further preclinical studies to validate the antiviral efficacy of *C. longa* bioactives and their potential applications in combating viral infections.

## 1 Introduction

*Curcuma longa*, commonly known as turmeric, is a flowering plant of the Zingiberaceae family, native to South Asia. For millennia, it has been cultivated for its rhizomes, rich in bioactive compounds widely used in traditional medicine, culinary applications, and as a natural dye. Curcumin, the principal bioactive compound in turmeric, makes up approximately 2%–5% of its weight and is responsible for its distinctive yellow color and numerous health benefits. The therapeutic relevance of *Curcuma longa* is attributed to its diverse phytochemical profile, which includes curcuminoids, essential oils, and polysaccharides. Among these, curcumin, demethoxycurcumin, and bisdemethoxycurcuminare the primary curcuminoids, with curcumin being the most studied for its potent antioxidant and anti-inflammatory properties (Krishnaiah et al., 2011; Hahn et al., 2020). Curcumin’s ability to scavenge reactive oxygen species (ROS) and inhibit pro-inflammatory cytokines like tumor necrosis factor-alpha (TNF-α) and interleukin-6 (IL-6) underlines its therapeutic significance (Sharifi-Rad et al., 2020).

Studies further underscore curcumin’s potential to reduce oxidative stress and inflammation (Miquel et al., 2002). For instance, Basak et al. (2020) demonstrated that curcumin significantly reduces lipid peroxidation and oxidative stress markers by approximately 40%. In addition to its anti-inflammatory properties, curcumin exhibits antiviral activity, affecting multiple stages of viral life cycles, from viral entry to genome replication and transcription. These properties highlight curcumin’s versatility in addressing a range of health issues, particularly inflammation and viral infections (Garodia et al., 2023). The recent outbreak of Mpox (Martín-Delgado et al., 2022; Ahmed et al., 2023), which the World Health Organization (WHO) declared a public health emergency in July 2022 with over 85,000 reported cases globally (Wei et al., 2022), has spurred further interest in turmeric’s medicinal properties. Given its well-documented therapeutic effects, *C. longa* is emerging as a potential natural remedy for treating viral infections like Mpox.

To evaluate the bioactive compounds in plant extracts, advanced analytical techniques such as High-Performance Liquid Chromatography (HPLC) and Thin-Layer Chromatography (TLC) are frequently employed. HPLC analysis has shown that curcumin constitutes approximately 36.33% of *C. longa* extracts, confirming its significant presence in the plant (Jyotirmayee and Mahalik, 2022). Additionally, computational approaches, such as molecular docking studies, are increasingly used to elucidate the mechanisms by which the phytochemicals in turmeric exert their therapeutic effects (Agamah et al., 2020). For instance, Nair and Paliwal (2021) reported that curcumin binds to key targets in inflammatory pathways, including nuclear factor-kappa B (NF-κB) and cyclooxygenase-2 (COX-2), both of which are triggered during viral infections. These insights are crucial for understanding how curcumin may serve as an antiviral agent, particularly in combating viral infections like Mpox (Pal et al., 2017). Molecular docking simulations have even predicted a binding affinity of −7.5 kcal/mol for curcumin with COX-2, suggesting strong interactions and therapeutic potential.

The aim of this study was to evaluate the therapeutic potential of an aqueous extract of *C. longa* (*C. longa*) against Mpox, with a focus on its antioxidant and anti-inflammatory properties. Inflammation is a common response to viral infections, often triggered by oxidative stress (Anyasor et al., 2019). To optimize the yield of bioactive compounds, particularly curcumin, the decoction method was employed for extraction. The biological activities of the extract were then investigated using various analytical techniques, including TLC and HPLC, which facilitated the quantification of key phytochemicals and provided a comprehensive framework for assessing the extract’s therapeutic efficacy. Unlike previous studies that focused solely on the antioxidant or anti-inflammatory properties of *C. longa*, this research uniquely integrates *in vitro* assays with *in silico* modeling to explore the molecular interactions between curcumin and Mpox virus proteins. The selection of Mpox protein targets for docking was based on their essential roles in the viral lifecycle, making them suitable candidates for therapeutic intervention. These proteins—4QVO, 8CEQ, and 8QRV—are involved in critical functions such as viral replication and mRNA modification, and their interaction with curcumin could provide insights into potential therapeutic strategies for treating Mpox (Elsheikh et al., 2023). This combined approach of biological assays and computational tools offers a more comprehensive evaluation of curcumin’s potential as a natural therapeutic agent, contributing new insights into its applicability for combating emerging infectious diseases like Mpox.

## 2 Experimental

**FIGURE 1:**
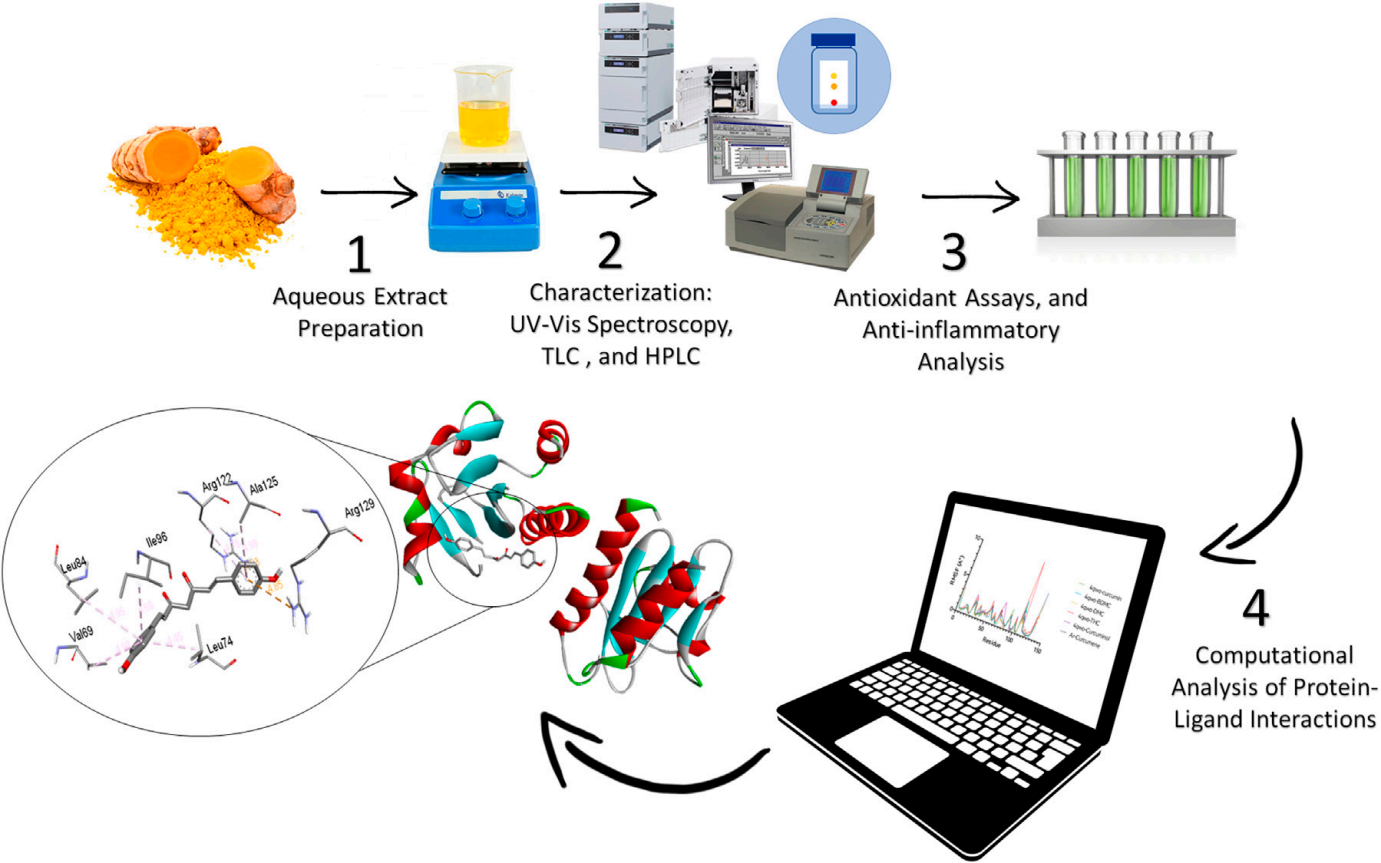
Experimental workflow of the extraction and computational analysis of aqueous Curcuma longa extract targeting Monkeypox.

Figure 1 illustrates the schematic representation of the experimental protocol used in this study. The process begins with the preparation of an aqueous extract from a natural source, such as turmeric, to isolate bioactive compounds. The extract is subsequently characterized using advanced analytical techniques, including UV-Vis spectroscopy, Thin Layer Chromatography (TLC), and High-Performance Liquid Chromatography (HPLC), to analyze its chemical composition. Following this, the biological activity of the extract is evaluated through *in vitro* assays to assess its antioxidant and anti-inflammatory properties. Finally, computational tools are utilized to study protein-ligand interactions, providing valuable insights into the binding affinity and mechanisms of action of the bioactive compounds.

### 2.1 Reagents and chemicals

All chemicals and reagents used in this study were of analytical grade and were supplied by Sigma-Aldrich unless otherwise specified. *C. longa* L. (turmeric) rhizomes were purchased in ground form. Methanol (CH₃OH) and chloroform (CHCl₃) were used as solvents for TLC and HPLC. Formic acid (HCOOH) was employed as a mobile phase additive during HPLC. Sulfuric acid (H₂SO₄), sodium phosphate (Na₃PO₄), and ammonium molybdate ((NH₄)₆Mo₇O₂₄) were purchased for the total antioxidant for the total antioxidant capacity (TAC) assay. Potassium ferricyanide (K₃[Fe(CN)₆]) and trichloroacetic acid (C₂HCl₃O₂) were used in th ferric reducing antioxidant power (FRAP) assay. All reagents were prepared fresh before use, and solvents used for extraction and chromatography were of HPLC grade.

### 2.2 Preparation of the C. longa aqueous extract (CAE)

To prepare the, 25 g of ground *C. longa* rhizomes was boiled in 250 mL of distilled water at 100°C in a reflux water bath for 15 min. The resulting mixture was filtered using Whatman No. 1 filter paper, and the filtrate was centrifuged at 4,000 rpm for 20 min. The supernatant was concentrated to dryness using a rotary evaporator (Heidolph Laborota 4,000), and the residue was stored in a refrigerator at 4°C until use (Serairi-Beji et al., 2018).

The extraction yield (Y) was calculated as follows:

$$Y\;\left(\%\right)=\text{mass of flask with extract }\left(g\right)-\text{mass of empty flask }\left(g\right)/\text{mass of the sample }\left(g\right))\times 100$$

### 2.3 UV-Vis spectroscopy analysis

The CAE maximum absorbance wavelength (λmax) was determined by scanning 1 mg/mL aqueous solution over a range of 400–800 nm using a UV-Vis-NIR spectrometer (JASCO V-770). Water was used as the blank, following the method described by Singh and Avupati (2017). To enhance the = measurement accuracy, data were acquired using the following parameters: scan speed of 200 nm/min, data interval of 1 nm, and slit width of 1 nm. Each measurement was taken in triplicate to ensure reproducibility, and the average absorbance values were recorded. The λmax value was identified as the wavelength at which the maximum absorbance occurred, indicating the most efficient light absorption by the active compounds present in the extract.

### 2.4 Thin-Layer Chromatography

TLC using Millipore Sigma TM silica gel plate analyzed the curcuminoids in the CAE. To enhance the separation efficiency, the silica gel plates were first activated by heating at 110°C for 30 min (Waksmundzka-Hajnos et al., 2022). The mobile phase comprised various ratios of methanol and chloroform (10:90, 25:75, 45:55, 50:50, 60:40, 75:25, and 90:10), prepared freshly for optimal separation. Uniform spots of the extract and of commercial controls (i.e., curcumin, demethoxycurcumin, bisdemethoxycurcumin), at equivalent concentrations, were applied approximately 1 cm from the plate bottom using a micropipette, plates were placed in a pre-saturated development chamber, allowing the solvent front to ascend for 8–10 cm. After the development phase, plates were removed, dried in a fume hood, and examined under UV light at 365 nm to visualize the spotsthatconfirmed the presence of curcuminoids by their characteristic fluorescence. The retention factor (Rf) of each component was calculated using the formula:

$$\text{Rf}=\left(\text{distance traveled by the sample }\left(h\right)\right)/\left(\text{distance traveled by the solvent front }\left(H\right)\right)$$

Each sample was analyzed in triplicate for reproducibility.

### 2.5 High-Performance Liquid Chromatography

To identify polyphenolic compounds, The CAE was filtered through a 0.45 µm membrane and injected into an Agilent Series 1100 HPLC system equipped with a multi-wavelength UV/Vis detector. A Kinetex column (100 × 4.6 mm, 2.6 µm) was used for separation. The mobile phase consisted of 0.1% formic acid in water and methanol, with a linear gradient elution from 5% to 95% methanol over 15 min. Detection was performed at 280 nm, and compounds were identified based on their retention times and spectral profiles compared with reference standards.

### 2.6 Total antioxidant capacity

The CAE was determined using the method described by Prieto et al. (1999) that, involves the formation of a green phosphate/Mo (V) complex. A reagent solution containing 0.3 N sulfuric acid, 28 mM sodium phosphate, and 4 mM ammonium molybdate was prepared. A 100 µL aliquot of diluted extract was mixed with 1 mL of reagent solution. After incubation in a boiling water bath for 90 min, the mixture was cooled, and absorbance was measured at 695 nm. Results were expressed as mg of gallic acid equivalent per Gram dry weight of the extract (mg GAE/g d.w.).

### 2.7 DPPH radical scavenging activity

The DPPH radical-scavenging activity was assessed using the method described by Sánchez-Moreno et al. (1998). Briefly, 50 µL of extract at different concentrations (0.078–5 mg/mL) was mixed with 1.95 mL of DPPH solution (0.025 g/L in methanol). After 30 min in the dark, absorbance was measured at 515 nm. Ascorbic acid (vitamin C) is used under the same conditions as a positive control. The IC_50_, the concentration required to inhibit 50% of DPPH radicals, was calculated as follows:

$$I\%=\left(A1-A2/\;A1\right)\times 100$$

Where I% is the percentage of inhibition, A1 is the absorbance of the control, and A2 is the absorbance in the presence of the extract.

### 2.8 Ferric reducing antioxidant power test

The FRAP test was performed according to Wu et al. (2014). CAE solutions (0.078–5 mg/mL) were mixed with 2.5 mL of 0.2 M phosphate buffer (pH 6.6) and 2.5 mL of 1% potassium ferricyanide. After incubation at 50°C for 20 min, 2.5 mL of 10% trichloroacetic acid was added, followed by centrifugation at 3,000 rpm for 10 min. The supernatant (2.5 mL) was mixed with 2.5 mL of distilled water and 0.5 mL of 0.1% FeCl₃ solution, and absorbance was measured at 700 nm. Ascorbic acid (vitamin C) is used under the same conditions as a positive control. The Antioxidant power was expressed as EC_50_ (i.e., the CAE concentration that causes the loss of 50% of the oxidant concentration).

### 2.9 Protein denaturation inhibition assay

The anti-inflammatory activity was evaluated using the albumin denaturation inhibition method, based on Nagavekar and Singhal (2018). A reaction mixture containing 1 mL of 1% egg or bovine serum albumin (BSA), 1 mL of PBS (pH 6.8), and 0.2 mL of the extract or standard drug (aspirin) was prepared and heated at 70°C for 5 min. After cooling, absorbance was measured at 660 nm. The percentage of albumin denaturation inhibition (I %) was calculated as follows:

$$I\%=\left(A1-A2/\;A1\right)\times 100$$

where A1 is the absorbance of the control and A2 is the absorbance of the test sample.

### 2.10 Molecular docking analysis

Potential ligands were selected among the bioactive compounds identified in the CAE by HPLC. The three-dimensional (3D) structures of these compounds were obtained from the PubChem database (pubchem.ncbi.nlm.nih.gov). The PDB IDs of the target proteins for this study were 4QWO (profiling-like protein), 8CEQ (cap-specific mRNA (nucleoside-2′-O-) - methyl transferase, and 8ORV (poxin-schlafen), which are critical enzymes involved in Mpox virus replication and pathogenicity. Their crystal structures were retrieved from the RCSB Protein Data Bank (www.rcsb.org). Enzyme preparation was performed using Molegro Virtual Docker 2.5, and molecular docking simulations were carried out with PyRx (version 0.8). The docking grid box parameters were: i) for 4QWO, the center coordinates were X: 3.3969, Y: 4.2924, Z: 14.3419, with dimensions of X: 52.8775 Å, Y: 59.5515 Å, and Z: 37.9783 Å; ii) for 8CEQ, the center coordinates were X: 21.8083 Å, Y: 25.8624 Å, and Z:12.8936, with dimensions of X: 56.8751 Å, Y: 51.9624 Å, and Z:31.5580 Å; andiii) for8ORV the center coordinates were X: 21.8083, Y: 25.8624, Z: 12.8936, with dimensions of X: 56.8751 Å, Y: 51.9624 Å, and Z: 31.5580 Å. The docking results were visualized with BIOVIA Discovery Studio Visualizer to provide detailed insights into the protein-ligand interactions, highlighting key hydrogen bonds and hydrophobic interactions between the ligands and the amino acid residues of the target proteins.

### 2.11 Molecular dynamic simulations

Molecular dynamics (MD) simulations were conducted using the GROMACS 2023-GPU package with the CHARMM-36–2019 force field to validate docking results and evaluate the dynamic stability of protein-ligand complexes (Boudou et al., 2024). The study focused on key monkeypox virus proteins, including 4QWO (profiling-like protein), 8CEQ (cap-specific mRNA (nucleoside-2′-O-) methyltransferase), and 8ORV (poxin-schlafen), which are crucial for viral replication and pathogenicity. Only the top-scoring protein-ligand complexes were selected for simulations. The systems were solvated in a cubic box with TIP3P water models, neutralized using Na^+^ and Cl^−^ ions, and subjected to energy minimization to eliminate steric clashes and optimize geometry. Subsequently, equilibration was carried out in two phases: constant volume (NVT ensemble) for 2 nanoseconds and constant pressure (NPT ensemble) for another 2 nanoseconds. Final production runs were conducted for 100 nanoseconds under constant temperature (300 K) and pressure (1 bar), enabling the assessment of stability and flexibility throughout the simulation.

### 2.12 Statistical analyses

All data are presented as mean ± SEM (Standard Error of the Mean). Statistical analyses were performed using Sigma Plot 11.0 for Windows. Comparisons between two means were evaluated using Student’s t-test, while comparisons among multiple means were conducted using Analysis of Variance (ANOVA) followed by *post hoc* tests recommended by the software, such as Student-Newman-Keuls, Bonferroni, or Tukey’s test. Significant differences between experimental groups are indicated by distinct letters (a-e), where groups not sharing the same letter are considered significantly different at *p* < 0.05.

## 3 Results and Discussion

### 3.1 Phytochemical analysis

**FIGURE 2:**
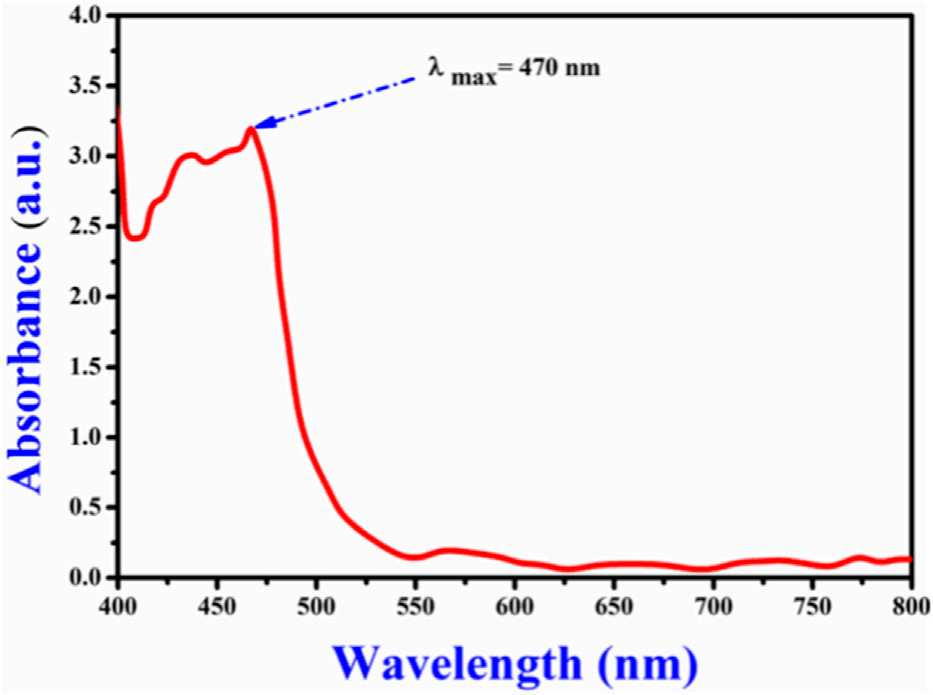
Maximum absorption wavelength (λmax) of the *Curcuma longa* aqueous extract, recorded at 470nm, indicating the presence of curcuminoids.

The CAE yield using the decoction method was 7.80% ± 0.81%, indicating the effective recovery of bioactive components from the plant material. The λmax of the CAE 470 nm (Figure 2), in agreement with the established λmaxrange (425–493 nm) for curcuminoids (Khumsupan et al., 2011; Sathishkumar et al., 2015). This finding supports the presence of curcuminoids, particularly curcumin, consistent with previous research (Pothitirat and Gritsanapan, 2006; de Oliveira Filho et al., 2021). The λmax value is essential for characterizing specific compounds and provides the foundation for the subsequent analytical and functional studies (Lozada-Ramírez et al., 2021).

**FIGURE 3:**
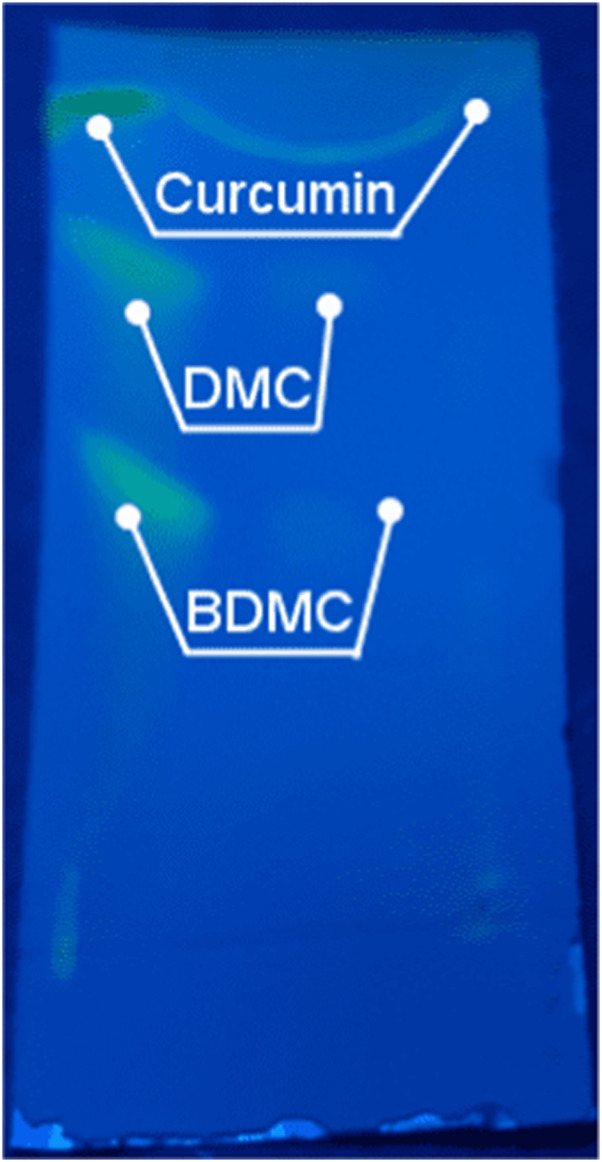
TLC separation of curcuminoid compounds from *Curcuma longa* aqueous extract, using a methanol/chloroform (90:10) solvent system. Distinct spots for curcumin, demethoxycurcumin (DMC), and bisdemethoxycurcumin (BDMC) were observed. Their Rf values are listed in Table 1.

**FIGURE 4:**
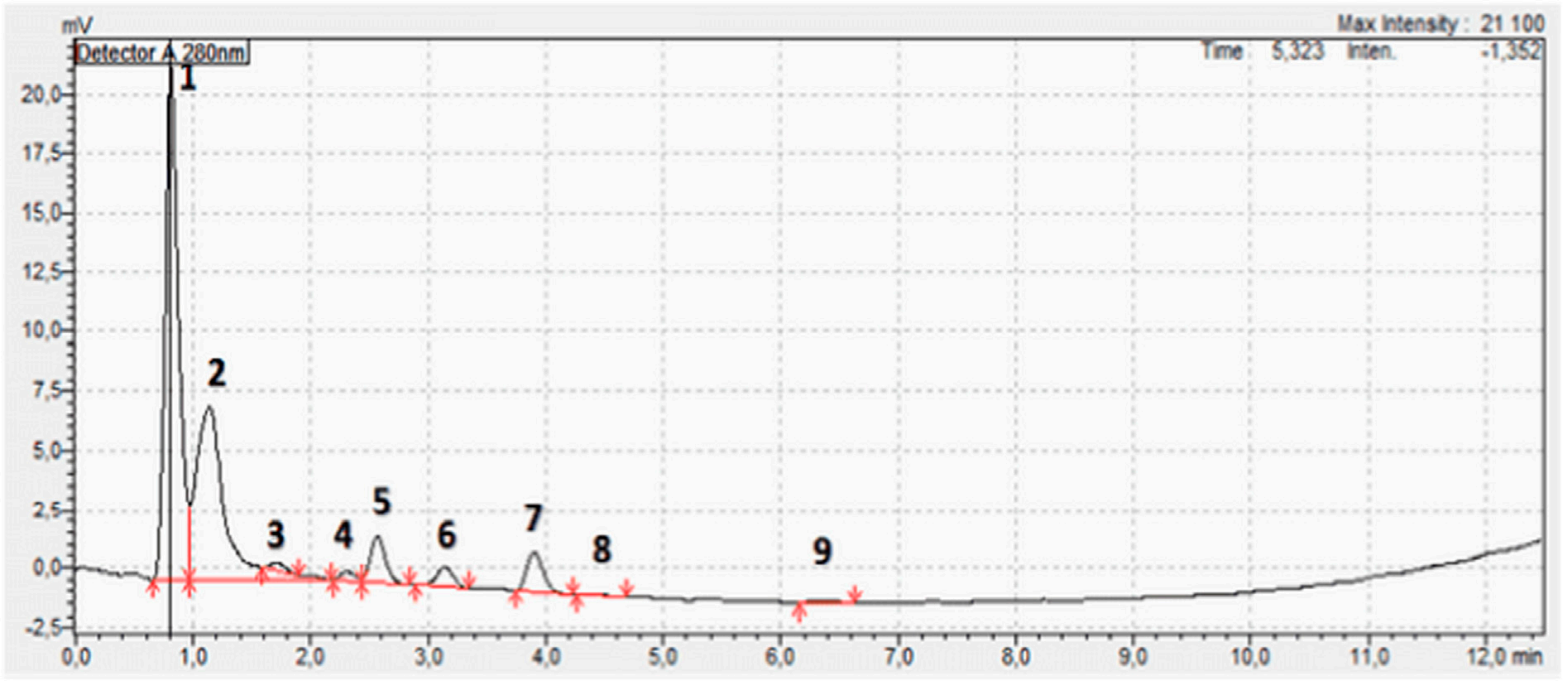
HPLC chromatogram of the *Curcuma longa* aqueous extract showing nine peaks. Seven compounds could be identified (Table 2) among which curcumin, (peak 2) was the most abundant (36.33%).

The TLC analysis successfully identified curcumin, demethoxycurcumin, and bisdemethoxycurcumin as the major curcuminoids in the extract (Figure 3). The observed Rf values (Table 1) were in line with previous reports, reinforcing TLC reliability as a preliminary phytochemical screening method (Sarwat et al., 2017). The HPLC analysis (Figure 4; Table 2) confirmed that curcumin was the predominant compound (36.33%), and that demethoxycurcumin, bisdemethoxycurcumin, and tetrahydrocurcuminwere present in smaller quantities (0.93%; 0.87%, 5.12%, respectively) in line with previous studies on *C. longa* extract phytochemical composition (Jyotirmayee and Mahalik, 2022). The identification of additional compounds, including curcuminol (2.39%) and Ar-curcumenone (4.92%), improves the understanding of *C. longa* chemical diversity, suggesting that these lesser-known constituents may contribute to its overall therapeutic efficacy. Moreover, the two unidentified compounds (0.32% and 0.33%) may represent additional bioactive substances, warranting additional investigations.

**TABLE 1:** Summary of the TLC results of curcuminoid compounds from *Curcuma longa* aqueous extract, using different methanol/chloroform solvent system. Distinct spots for curcumin, demethoxycurcumin (DMC), and bisdemethoxycurcumin (BDMC) were observed.

Samples	Mobile phase	Ratio	Rf values		
			Curcumin	DMC	BDMC
CAE	Methanol: chloroform	90: 10	0.95	0.71	0.55
Literature data (Sarwat et al., 2017)	Methanol: chloroform	98: 2	0.90	0.75	0.69

**TABLE 2:** HPLC analysis of the *Curcuma longa* aqueous extract: identified compounds and their proportions.

Peak#	Retention time (min)	Concentration (%)	Compound name
1	0.81	48.80	Solvent
2	1.14	36.33	Curcumin
3	1.71	0.87	Bisdemethoxycurcumin
4	2.31	0.93	Demethoxycurcumin
5	2.57	5.12	Tetrahydrocurcumin
6	3.14	2.39	Curcuminol
7	3.90	4.92	Ar-curcumene
8	4.41	0.32	Unidentified
9	6.35	0.33	Unidentified

The HPLC results confirmed that curcumin, recognized for its anti-inflammatory, antioxidant, and anticancer activities (Sengupta and Tripathi, 2023), was the major component. The presence of demethoxycurcumin (0.93%) and bisdemethoxycurcumin (0.87%), although in smaller quantities, enhances the extract potential because these compounds exhibit similar biological effects (Anand et al., 2008). Moreover, tetrahydrocurcumin, known for its potential neuroprotective effects, and curcuminol associated with improved bioavailability, enhance the extract therapeutic profile (Nile and Park, 2015; Fuloria et al., 2022).

### 3.2 *In Vitro* antioxidant activity

**FIGURE 5:**
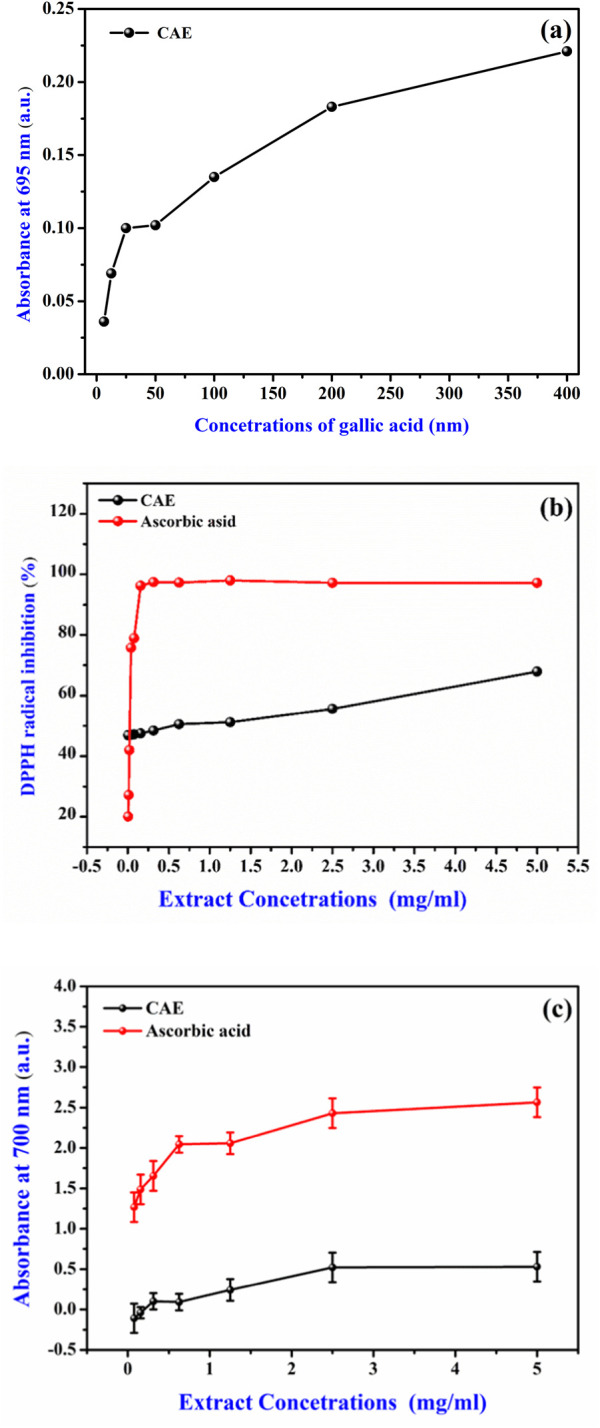
Antioxidant potential of the *Curcuma longa* aqueous extract (CAE) **(A)** Gallic acid calibration curve for total antioxidant activity, expressed as mg of gallic acid equivalent per Gram dry weight of the extract (mg GAE/g d.w.). **(B)** Antioxidant activity of CAE via DPPH scavenging compared to ascorbic acid (Vitamin C). **(C)** Ferric reducing antioxidant power (FRAP) of CAE compared to ascorbic acid (Vitamin C).

The *in vitro* antioxidant activity of the CAE was assessed using three assays: total antioxidant capacity, DPPH radical scavenging, and FRAP radical scavenging (Figure 5). The results, summarized in Table 3, demonstrated a substantial antioxidant potential of the CAE. Specifically, its TAC was 36.55 ± 0.01 µg GAE/g d.w., in line with previous reports highlighting the potent antioxidant properties of *C. longa*. extract, primarily attributed to the high content of bioactive compounds, particularly curcuminoids (Sabale et al., 2013). The TAC value reflects the CAE overall ability to neutralize free radicals, a crucial function for limiting oxidative stress and preventing cellular damage.

**TABLE 3:** Total antioxidant activity, DPPH radical scavenging, and ferric reducing power of CAE.

Extract	TAC (µg GAE/g d.w.)	IC_50_ of DPPH (mg/mL)	EC_50_ of FRAP (mg/mL)
CAE	36.55 ± 0.01	0.77 ± 0.04^a^	3.46 ± 0.11^a^
Ascorbic Acid	NT	0.019 ± 0.001^b^	0.005 ± 0.003^b^

CAE: aqueous curcuma longa extract; TAC: total antioxidant activity; IC50: Median inhibitory concentration; EC50: Median effective concentration; NT: Not tested. Data are expressed as mean ± SEM (n = 3). Group comparisons were made using Tukey’s test. Columns with different letters (a-b) are significantly different at *p* < 0.05 (Tukey’s test).

In the DPPH radical scavenging assay, the CAE IC_50_ value was 0.77 ± 0.04 mg/mL, indicating its effective capacity to scavenge free radicals. The IC_50_ value was comparable to that ofother plant extracts rich in phenolic and flavonoid compounds, which are known for their free radical scavenging activity (Pereira et al., 2018). The presence of curcumin and other curcuminoids, such asdemethoxycurcumin and bisdemethoxycurcumin, likely contributes to this activity. It is well known that curcumin can donate hydrogen atoms and neutralize free radicals, reinforcing the observed scavenging efficacy (Aggarwal et al., 2007). Studies have shown that curcumin exhibits the highest antioxidant activity, followed by demethoxycurcumin and bisdemethoxycurcumin, confirming the significant role of these curcuminoids in free radical scavenging (Jayaprakasha et al., 2006). Additionally, demethoxycurcumin and bisdemethoxycurcumin have been found to enhance the antioxidant activity of curcumin when tested in refined sunflower oil, further demonstrating their synergistic effect in antioxidant mechanisms (Rege et al., 2014). Moreover, hydrogenated derivatives of curcumin, such as tetrahydrocurcumin, have been shown to exhibit even stronger antioxidant activities (Somparn et al., 2007).

The FRAP assay result (EC_50_ value of 3.46 ± 0.11 mg/mL) further supports the CAE antioxidant profile. This test evaluates the electron-donating capacity of an extract that is essential for breaking free radical chains and preventing oxidative damage. The CAE reducing power indicates its ability to act as an electron donor, converting ferric ions (Fe³⁺) into ferrous ions (Fe^2^⁺), a key mechanism in the antioxidant defense. This activity is consistent with the properties of curcuminoids, and other polyphenolic compounds found in *C. longa* that display strong electron-donating ability (Wang et al., 2017). Although the extract shows lower activity than ascorbic acid, its significant antioxidant potential indicates its promise as a natural alternative for further development and synergistic applications.

### 3.3 *In Vitro* anti-inflammatory activity

The CAE *in vitro* anti-inflammatory activity was evaluated by measuring the inhibition of heat-induced protein denaturation, using egg albumin and BSA (Chandra et al., 2012). The maximum protein denaturation inhibition values 78.88% ± 0.53% for egg albumin and 90.51% ± 0.29% for BSA were obtained with 5,000 μg/mL CAE (Table 4) indicating significant anti-inflammatory potential. The *in vitro* anti-inflammatory activity of the *C. longa* aqueous extract was evaluated by measuring its inhibition of heat-induced protein denaturation, using egg albumin and BSA as models. The maximum inhibition values were 78.88% ± 0.53% for egg albumin and 90.51% ± 0.29% for BSA at a concentration of 5,000 μg/mL CAE (Table 4), demonstrating significant anti-inflammatory potential. This result aligns with findings from Bagad et al. (2013), who also reported notable anti-inflammatory effects of an aqueous extract of *C. longa* in both acute and chronic inflammation models. Additionally, curcumin, the primary bioactive compound in *C. longa*, is well-known for its ability to inhibit key pro-inflammatory pathways, as detailed by Peng et al. (2021), which underscores its effectiveness in treating various inflammation-related diseases.

**TABLE 4:** Anti-inflammatory activity of the *Curcuma longa* aqueous extract (CAE), at different concentrations, and aspirin.

Samples	Concentration (µg/mL)	Egg albumin denaturationinhibition (%)	BSA denaturationinhibition (%)
CAE	5,000	78.88 ± 0.53^a^	90.51 ± 0.29^b^
CAE	2,500	70.12 ± 0.43^a^	89.41 ± 0.05^b^
CAE	1,250	65.34 ± 0.85^a^	89.00 ± 0.10^b^
CAE	625	59.36 ± 0.27^a^	79.50 ± 0.29^b^
CAE	313	52.59 ± 0.49^a^	73.87 ± 0.22^b^
CAE	157	45.82 ± 0.22^a^	71.48 ± 0.13^b^
Aspirin	1,000	88.80 ± 0.28^a^	95.37 ± 0.23^b^

Data isthe mean ± SD (n = 3).

Aspirin (acetylsalicylic acid) used as the reference anti-inflammatory compound, demonstrated higher protein denaturation inhibition rates at 1,000 μg/mL, with values of 88.80% ± 0.28% for egg albumin and 95.37% ± 0.23% for BSA. In comparison, the inhibition effects of the *C. longa* aqueous extract decreased at lower concentrations, showing a clear dose-dependent response. For instance, at 625 μg/mL, the extract inhibited egg albumin and BSA denaturation by 59.36% ± 0.27% and 79.50% ± 0.29%, respectively. This indicates that the anti-inflammatory potential of CAE is concentration-dependent, with higher doses providing stronger inhibition. Although the exact mechanism by which CAE inhibits protein denaturation remains to be fully understood, research by Chen et al. (2022) offers insight into how certain phenolic compounds, such as tannic acid, epigallocatechin gallate, quercetin, and quercitin, interact with proteins like casein. These phenolic compounds promote protein aggregation, enhance emulsifying and foaming stability, and alter protein structure. Their interactions are influenced by factors such as molecular weight, hydroxyl group positioning, acylation, and glycosylation, which affect protein behavior. It is possible that similar interactions involving tannins in CAE protect proteins from heat-induced damage by forming chemical bonds that stabilize the protein structure. This stabilization may reduce the entropy of the polypeptide chain, slowing down the denaturation process and providing a protective effect.

These findings also align with previous research on the anti-inflammatory effects of *C. longa* extracts, supporting its traditional use in managing inflammatory conditions (Koosirirat et al., 2010; Bagad et al., 2013). The inhibition of albumin denaturation suggests that *C. longa* aqueous extract (CAE) could serve as a natural alternative to conventional anti-inflammatory drugs like aspirin, providing therapeutic benefits with potentially fewer side effects. The effectiveness of CAE in inhibiting protein denaturation is largely attributed to its active compounds, primarily curcumin and its derivatives. Curcumin’s anti-inflammatory properties are well-documented, and its mechanism of action includes the inhibition of key enzymes, such as cyclooxygenases (COX) and lipoxygenases (LOX), which are involved in the production of pro-inflammatory mediators like prostaglandins and leukotrienes. Additionally, curcumin suppresses the activity of pro-inflammatory cytokines, including TNF-α, IL-1β, and IL-6, which are central to the inflammatory process. Its ability to inhibit the NF-κB signaling pathway, a critical regulator of inflammation, further supports curcumin’s role in reducing the transcription of genes linked to inflammation (Kunnumakkara et al., 2017; Gupta et al., 2013). These combined mechanisms highlight *C. longa*’s potential as an effective natural anti-inflammatory agent.

### 3.4 Molecular docking to assess the interaction between CAE compounds and Mpoxvirus protein

**FIGURE 6:**
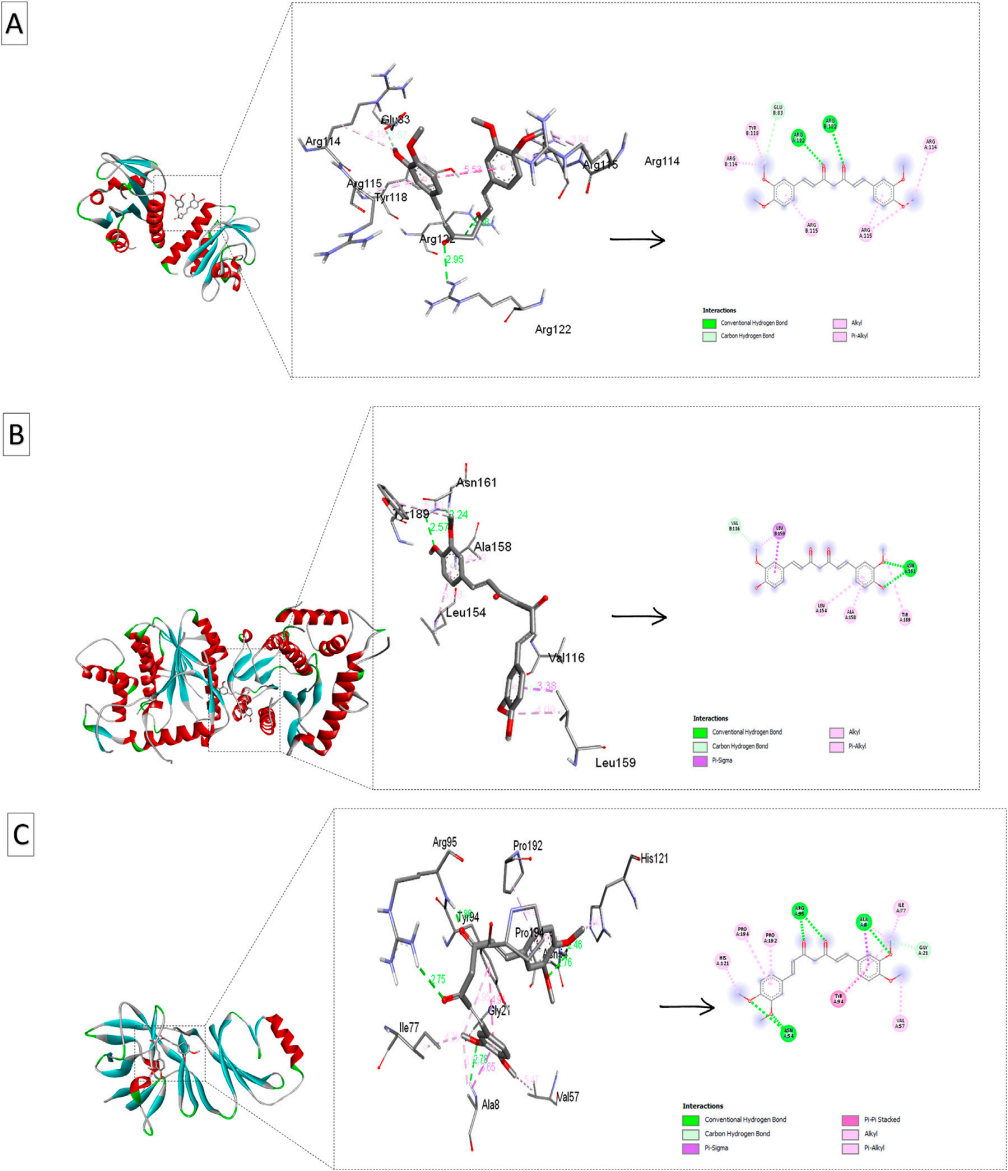
Ribbon views and active site interactions of demethoxycurcumin and curcumin with the target proteins. Ribbon views (left) and active site details (left) highlighting the ligand-enzyme interactions (right). **(A)** Demethoxycurcumin complexed with 4QWO. **(B)** Curcumin complexed with 8CEQ. **(C)** Demethoxycurcumin complexed with 8ORV.

The molecular docking results (Table 5; Figure 6) offer insights into the interaction between curcumin derivatives and three target proteins (PDB IDs: 4QWO, 8CEQ, and 8ORV) implicated in Mpox virus replication and pathogenicity. These interactions revealed the binding affinities, and the residues involved in ligand-target protein interactions, suggesting how these compounds could inhibit specific biological pathways involved in viral infection.

**TABLE 5:** Binding affinity and molecular interactions of ligands identified in the *Curcuma longa* aqueous extract with target Mpox virus proteins.

Protein	Ligand (ID PubChem)	Estimated free energy of binding (kcal/mol)	Residues involved in bonded interactions
PDB ID: 4QWO	Curcumin (ID:969516)	−6.7	Arg B: 114, Arg B: 115, Tyr B: 118, Tyr A: 118, Arg B: 119, Arg A: 119, Arg A:122, Arg B:122
	Bisdemethoxycurcumin (ID: 5315472)	−6.5	Ala B: 125, Arg B: 129, Arg B: 122, Leu B: 74, Ile B: 96, Leu B: 84, Val B: 69
	Demethoxycurcumin (ID: 9952605)	−7.7	Arg B: 114, Arg A: 114, Arg B: 115, Arg A: 115, Arg B: 122, Arg A: 122, Tyr B: 118, Glu B: 83
	Tetrahydrocurcumin (ID:124072)	−6.6	Arg A: 115, Arg B: 115, Arg B: 122, Arg A: 112, Tyr B: 118
	Curcuminol (ID:169436751)	−6.4	Arg B: 114, Tyr B: 118, Arg A: 115, Arg B: 115, Arg A: 122
	Ar-curcumene (ID:3083834)	−6.3	Arg A: 115, Arg B: 115, Tyr A: 118, Arg A: 119, Arg B: 119
PDB ID: 8CEQ	Curcumin (ID:969516)	−8.1	Val B: 116, Leu B: 159, Leu A: 154, Ala A: 158, Tyr A: 189, Asn A: 161
	Bisdemethoxycurcumin (ID: 5315472)	−7.9	Gln A: 39, Leu A: 42, Arg A: 97, Phe A: 115, Val A: 116, Val A: 139, Arg A: 140
	Demethoxycurcumin (ID: 9952605)	−7.8	Gly B: 68, Ala B: 70, Gly B: 96, Arg B: 97, Asp B: 95, Asp B: 138, Phe A: 188, Tyr A: 189
	Tetrahydrocurcumin (ID:124072)	−7.3	Asp B: 95, Gly B: 96, Arg B: 97, Phe B: 115, Val B: 116, Val B: 139, Asn A: 161, Tyr A: 189, Ala A: 158, Leu B: 159
	Curcuminol (ID:169436751)	−8.0	Gly B: 96, Arg B: 140, Ser B: 141, Tyr A: 189, Asn A: 161
	Ar-curcumene (ID:3083834)	−6.7	Gly B: 96, Val B: 116, Phe B: 115, Ala A: 158, Leu B: 159, Phe A: 188
PDB ID: 8ORV	Curcumin (ID:969516)	−7.1	Ala A: 8, Gly A: 21, Ile A: 77, Asn A: 54, Thr A: 93, Tyr A: 94, Arg A: 95, Pro A: 192, Pro A: 194
	Bisdemethoxycurcumin (ID: 5315472)	−6.8	Ala A: 8, Asn A: 54, Thr A: 93, Tyr A: 94, Arg A: 95
	Demethoxycurcumin (ID: 9952605)	−7.4	Ala A: 8, Gly A: 21, Asn A: 54, Val A: 57, Ile A: 77, Tyr A: 94, Arg A: 95, His A: 121, Pro A: 192, Pro A: 194
	Tetrahydrocurcumin (ID:124072)	−6.3	Asn A: 54, Ser A: 55, Gln A: 56, Tyr A: 94, Pro A: 192, Pro A: 194
	Curcuminol (ID:169436751)	−7.0	Ala A: 8, Tyr A: 94, Asn A: 54, Ser A: 55, Gln A: 56, Arg A: 95, His A: 121, Pro A: 192, Ile A: 193, Pro A: 194
	Ar-curcumene (ID:3083834)	−5.8	Ile A: 77, Tyr A: 94, His A: 121, Leu A: 191, Pro A: 192, Pro A: 194

4QWO:demethoxycurcuminshowed the strongest binding affinity (−7.7 kcal/mol), and interacted with Arg B: 114, Arg B: 115, and Glu B: 83. This high affinity indicates strong molecular interactions with the target virus protein, suggesting that demethoxycurcuminmay have a significant inhibitory effect on the targeted pathway. Curcumin had a slightly lower binding affinity (−6.7 kcal/mol) and alsointeracted with key residues (Arg B: 114, Arg B: 115, Tyr B: 118), indicating that it retains substantial efficacy.

8CEQ: Curcumin exhibited the highest binding affinity (−8.1 kcal/mol) and interacted with Val B: 116 and Tyr A: 189. This suggests that curcumin can strongly bind to the active site of this protein, potentially inhibiting its function. The strong affinity of bisdemethoxycurcumin (−7.9 kcal/mol) and demethoxycurcumin (−7.8 kcal/mol) and interaction with key residues in 8CEQ further support their role as effective anti-inflammatory/antiviral agents.

8ORV:Demethoxycurcumin again showed the strongest binding affinity (−7.4 kcal/mol) and, interacted with Ala A: 8 and Gly A: 21, highlighting its broad potential across different target proteins. Curcumin and curcuminol (-7.1 kcal/mol and −7.07 kcal/mol respectively), also showed significant interactions with key residues.

In a related study, Mulu et al. (2021) demonstrated that curcumin-derived polyphenols, specifically demethoxycurcumin and bisdemethoxycurcumin, exhibited significant binding affinities to the COVID-19 main protease, indicating their potential as effective antiviral agents. The therapeutic effects of these curcumin derivatives have been extensively researched, particularly concerning inflammation and oxidative stress-related diseases. Recent literature indicates that demethoxycurcumin and bisdemethoxycurcumin modulate various molecular targets, including inflammatory enzymes like COX-2, transcription factors such as NF-κB, and cytokines like TNF-α and IL-6 (Bahuguna et al., 2021). The molecular docking results from this study further substantiate the ability of curcumin derivatives to interact with specific amino acid residues within the active sites of viral proteins, which may play a role in inflammatory responses. Notably, demethoxycurcumin exhibits superior binding affinity, suggesting that the absence of one methoxy group enhances its interactions with target proteins, thereby increasing its efficacy compared to curcumin.

This finding also aligns with previous reports indicating that demethoxycurcumin demonstrates higher biological activity than curcumin in anti-inflammatory assays. Across all protein structures tested, demethoxycurcumin consistently showed the strongest binding affinities, suggesting it may be the most potent ligand among the curcumin derivatives. Its interactions with critical residues, such as Arg, Tyr, and Glu, in the active sites of these proteins may account for its enhanced therapeutic effects. These molecular docking results are consistent with experimental studies demonstrating that demethoxycurcumin effectively modulates inflammatory responses by interacting with relevant enzymes and signaling pathways.

### 3.5 Molecular dynamic simulations

The molecular dynamics (MD) simulations of the three selected complexes, 8CEQ-Curcumin, 4QVO-DMC, and 8QRV-DMC, were performed to evaluate their stability, compactness, and residue flexibility. These complexes were chosen because they represented the best docking results, exhibiting the highest binding affinities and optimal interactions with the target protein during the molecular docking analysis.

**FIGURE 7:**
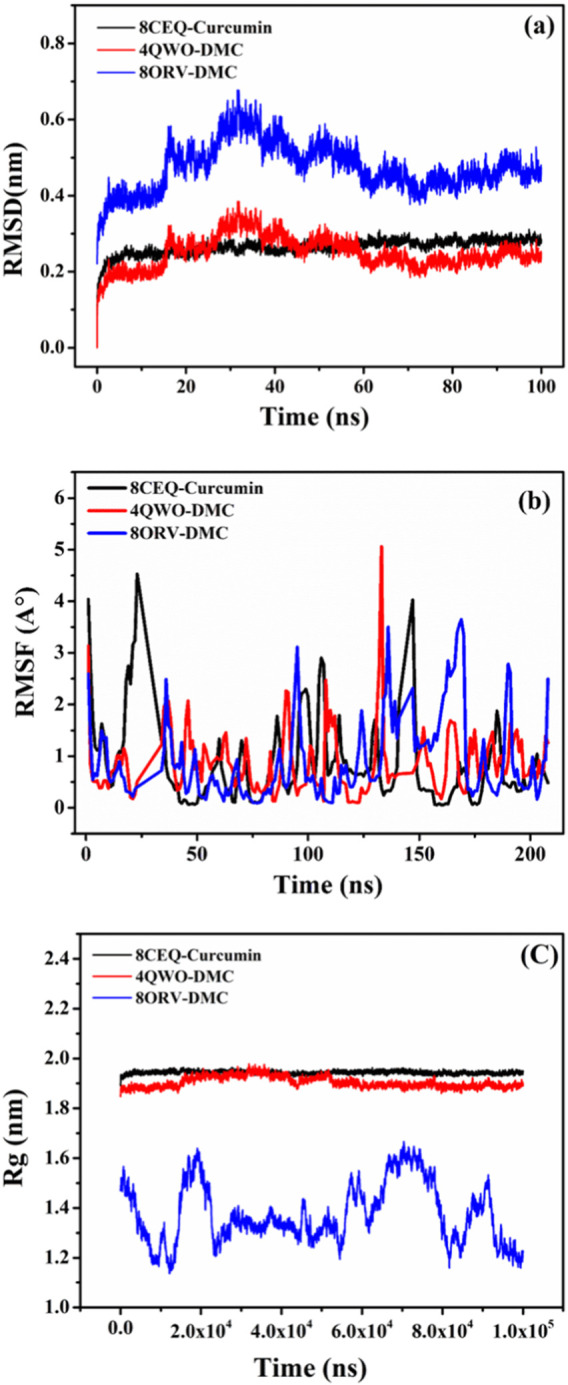
MD simulation results for the three protein-ligand complexes: **(A)** RMSD analysis of the 8CEQ-Curcumin, 4QVO-DMC, and 8QRV-DMC complexes over time, indicating the stability of the complexes during the simulation. **(B)** RMSF analysis of the 8CEQ-Curcumin, 4QVO-DMC, and 8QRV-DMC complexes over time, demonstrating the flexibility of the protein-ligand interactions at specific residues. **(C)** Rg analysis of the 8CEQ-Curcumin, 4QVO-DMC, and 8QRV-DMC complexes over time, reflecting the compactness and stability of the molecular complexes.

The RMSD analysis (Figure 7A) showed that all three complexes reached equilibrium during the simulation period, with deviations ranging between 0.2 and 0.6 nm. Among them, 4QVO-DMC exhibited the lowest fluctuations, indicating greater stability compared to the other two complexes. The 8CEQ-Curcumin complex showed slightly higher fluctuations during the middle phase of the simulation but stabilized toward the end, whereas the 8QRV-DMC complex maintained relatively moderate fluctuations throughout the simulation.

The Rg analysis (Figure 7C) provided insights into the compactness of the complexes over time. All three complexes displayed consistent Rg values between 1.8 and 2.0 nm, suggesting stable and compact structures throughout the simulation. Notably, 4QVO-DMC exhibited the most consistent compactness, further emphasizing its stability. In contrast, 8CEQ-Curcumin and 8QRV-DMC showed slightly more variability, with occasional increases in Rg, indicating minor structural rearrangements during the simulation.

The RMSF analysis (Figure 7B) evaluated the flexibility of individual residues in the protein-ligand complexes. Most residues displayed minimal fluctuations, generally below 1 nm, indicating structural rigidity in all complexes. However, higher fluctuations were observed at terminal and loop regions, which are typically more flexible. The 8CEQ-Curcumin complex exhibited higher residue fluctuations at specific regions compared to the other two complexes. The 4QVO-DMC complex showed the least residue fluctuations, while the 8QRV-DMC complex exhibited intermediate flexibility, suggesting a balance between stability and dynamic behavior.

The observations of stability and dynamic behavior align with previous studies, such as the work by Dutt et al. (2023), which investigated the stability of small molecule inhibitors targeting DNA-dependent RNA polymerase (DdRp) in Mpox virus through MD simulations. In their study, the top-ranked complexes, including lumacaftor and conivaptan, were subjected to 200 ns of MD simulations and principal component analysis (PCA), which revealed highly stable interactions of these inhibitors with DdRp. The high stability observed in these drug-target complexes underscores the value of MD simulations in validating docking results and understanding the dynamic interplay between ligands and their target proteins.

These findings align with studies such as Dutt et al. (2023), which explored the stability of small molecule inhibitors targeting DNA-dependent RNA polymerase (DdRp) in monkeypox virus. In their MD simulations, stable interactions were observed between top-ranked inhibitors and DdRp, highlighting the importance of MD simulations in validating docking results and understanding protein-ligand dynamics. Similarly, Mulu et al. (2021) found that curcumin-derived polyphenols exhibit variations in flexibility and binding affinity when interacting with target proteins, emphasizing the role of dynamic regions in protein-ligand interactions. Despite fluctuations, key residues such as Gln817, His613, Asp764, and His617 remained stable in all three complexes, indicating that flexibility does not disrupt specific interactions, aligning with Tuffery and Derreumaux (2012).

The 8CEQ-Curcumin complex, although stable overall, displayed more flexibility, suggesting less robust binding compared to the DMC complexes. In contrast, both 4QVO-DMC and 8QRV-DMC exhibited greater stability and compactness, consistent with Rampogu et al. (2021), which demonstrated that curcumin analogues, including demethoxycurcumin (DMC), exhibit stable interactions and balanced flexibility in MD simulations. The 4QVO-DMC complex, in particular, demonstrated superior stability, making it the most promising candidate for further investigation. The 8QRV-DMC complex also displayed stable interactions but with slightly higher flexibility compared to 4QVO-DMC. These results highlight DMC’s potential as a more stable curcumin derivative, consistent with Maiti et al. (2021), which showed that DMC retains strong binding stability and therapeutic potential *in silico* analyses.

## 4 Conclusion

In conclusion, this study provides a comprehensive analysis of a *C. longa* decoction extract revealed an extraction yield of 7.80% ± 0.81%, in which curcumin was the predominant compound (36.33%), underscoring its therapeutic significance. The presence of demethoxycurcumin (0.93%) and bisdemethoxycurcumin (0.87%) enhances the extract’s therapeutic potential because these compounds exhibit complementary biological effects. The λmax of 470 nm confirmed the presence of curcuminoids and serves as a benchmark for standardization in future studies. The TLC results validated the extraction methodology, and HPLC allowed identifying additional compounds, including tetrahydrocurcumin and curcuminol, that may further contribute to the extract’s therapeutic efficacy. The notable antioxidant capacity (36.55 ± 0.01 mg GAE/g w.d.) supports its traditional use against oxidative stress. Moreover, the extract’s significant inhibition of heat-induced albumin denaturation highlights its anti-inflammatory potential, suggesting applications in inflammatory diseases.The molecular docking and dynamics simulations offer valuable insights into the interactions between curcumin derivatives and monkeypox virus proteins, revealing demethoxycurcumin’s superior binding affinity and stability. Overall, this study enhances our understanding of *C. longa's* phytochemical profile and molecular behavior, advocating further research on its pharmacological applications and the synergistic effects of its various bioactive constituents. The combination of experimental and computational approaches strengthens the case for *C. longa* as a valuable resource for therapeutic applications, particularly in the context of chronic diseases characterized by inflammation and oxidative stress.

## Data Availability

The data supporting the findings of this study will be made available upon reasonable request.
